# Characterization of kinase gene expression and splicing profile in prostate cancer with RNA-Seq data

**DOI:** 10.1186/s12864-018-4925-1

**Published:** 2018-08-13

**Authors:** Huijuan Feng, Tingting Li, Xuegong Zhang

**Affiliations:** 1 MOE Key Laboratory of Bioinformatics, Division of Bioinformatics and Center for Synthetic and Systems Biology, TNLIST, Department of Automation, Tsinghua University, Beijing, 100084 China; 2 Department of Biomedical Informatics, Institute of Systems Biomedicine, School of Basic Medical Sciences, Peking University Health Science Center, Beijing, 100191 China; 3 School of Life Sciences, Tsinghua University, Beijing, 100084 China; 4 Present address: Department of Systems Biology, Department of Biochemistry and Molecular Biophysics, Center for Motor Neuron Biology and Disease, Columbia University, New York, NY 10032 USA

**Keywords:** Prostate cancer, Alternative splicing, Kinase, CDK5, Isoform switching

## Abstract

**Background:**

Alternative splicing is a ubiquitous post-transcriptional regulation mechanism in most eukaryotic genes. Aberrant splicing isoforms and abnormal isoform ratios can contribute to cancer development. Kinase genes are key regulators of multiple cellular processes. Many kinases are found to be oncogenic and have been intensively investigated in the study of cancer and drugs. RNA-Seq provides a powerful technology for genome-wide study of alternative splicing in cancer besides the conventional gene expression profiling. But this potential has not been fully demonstrated yet.

**Methods:**

We characterized the transcriptome profile of prostate cancer using RNA-Seq data from viewpoints of both differential expression and differential splicing, with an emphasis on kinase genes and their splicing variations. We built a pipeline to conduct differential expression and differential splicing analysis, followed by functional enrichment analysis. We performed kinase domain analysis to identify the functionally important candidate kinase gene in prostate cancer, and calculated the expression levels of isoforms to explore the function of isoform switching of kinase genes in prostate cancer.

**Results:**

We identified distinct gene groups from differential expression and splicing analyses, which suggested that alternative splicing adds another level to gene expression regulation. Enriched GO terms of differentially expressed and spliced kinase genes were found to play different roles in regulation of cellular metabolism. Function analysis on differentially spliced kinase genes showed that differentially spliced exons of these genes are significantly enriched in protein kinase domains. Among them, we found that gene CDK5 has isoform switching between prostate cancer and benign tissues, which may affect cancer development by changing androgen receptor (AR) phosphorylation. The observation was validated in another RNA-Seq dataset of prostate cancer cell lines.

**Conclusions:**

Our work characterized the expression and splicing profiles of kinase genes in prostate cancer and proposed a hypothetical model on isoform switching of CDK5 and AR phosphorylation in prostate cancer. These findings bring new understanding to the role of alternatively spliced kinases in prostate cancer and also demonstrate the use of RNA-Seq data in studying alternative splicing in cancer.

**Electronic supplementary material:**

The online version of this article (10.1186/s12864-018-4925-1) contains supplementary material, which is available to authorized users.

## Background

Alternative splicing is an important post-transcriptional regulation mechanism through which one gene can produce multiple isoforms. It is ubiquitous in human cells, and about 95% of human multi-exon genes undergo this process [1]. The multiple protein isoforms generated with this mechanism play critical roles in diverse cellular processes such as cell cycle control, differentiation and cell signaling, etc. [2]. Aberrant alternative splicing has been reported to be highly relevant to many human diseases including cancer. Many cancer-related genes undergo alternative splicing and cancer-specific alternative splicing events have been found to contribute to carcinogenesis [3]. RNA-Seq technology and bioinformatics methods have provided great opportunities for studying alternative splicing in cancers [4].

Prostate cancer is a major type of cancer in men. Normal prostate tissue development needs steady activity of androgens with androgen receptors (AR). AR is an important transcription factor that regulates the expression of many downstream genes. AR transcriptional activity, i.e., its regulatory activity on downstream genes, is modulated by interactions of AR and co-regulators. Some kinase-related signal transduction pathways can regulate AR transcriptional activities via phosphorylation of AR and AR co-regulators, which may be a main mechanism to maintain AR transcriptional activity in AR-negative prostate cancer cells [5]. Protein kinases are one of the largest gene families in human and they constitute ~ 1.7% of human genes [6]. Mutations and dysregulation of protein kinases have been found to be causal for some human diseases, and especially for cancer. Efforts have been made to target oncogenic kinases by developing inhibitors for disease therapy [7]. Merkin et al. revealed that a large percentage of alternative splicing events often contribute to alterations in protein phosphorylation and kinase signaling [8]. It has been observed that some aberrant splicing events can modify kinase activities by truncating the kinase domain or fine-tuning the binding specificity to functional partners [9].

Many efforts have been devoted to the study of kinases that are deregulated in cancer and may serve as potential targets for cancer treatment. Differential expression of kinase genes in prostate cancer has been studied with RT-PCR [10] and microarrays [11], but the alternative splicing of these genes have not been systematically studied in prostate cancer. In this study, using an RNA-Seq dataset published by Kannan et al. [12], we characterized the transcriptome profile in prostate cancer on both differential expression and differential splicing, with emphases on kinase genes and their splicing isoforms. We profiled genes that are differentially expressed (DE) and differentially spliced (DS) between prostate cancer and benign tissues, and used GO and KEGG pathway enrichment analyses to reveal their distinct functions in prostate cancer. We studied the kinases among the detected genes and identified kinases that may have function alterations caused by differential splicing through protein domain analysis. Among them, CDK5 was detected to undergo isoform switching between prostate cancer and benign tissues, which suggests an important regulatory role of CDK5 alternative splicing on AR phosphorylation in prostate cancer. The result was validated on another RNA-Seq dataset of prostate cancer cell lines [13], and also has implications regarding the difference between androgen-dependent and androgen-independent cancer progressions.

## Methods

**Fig. 1:**
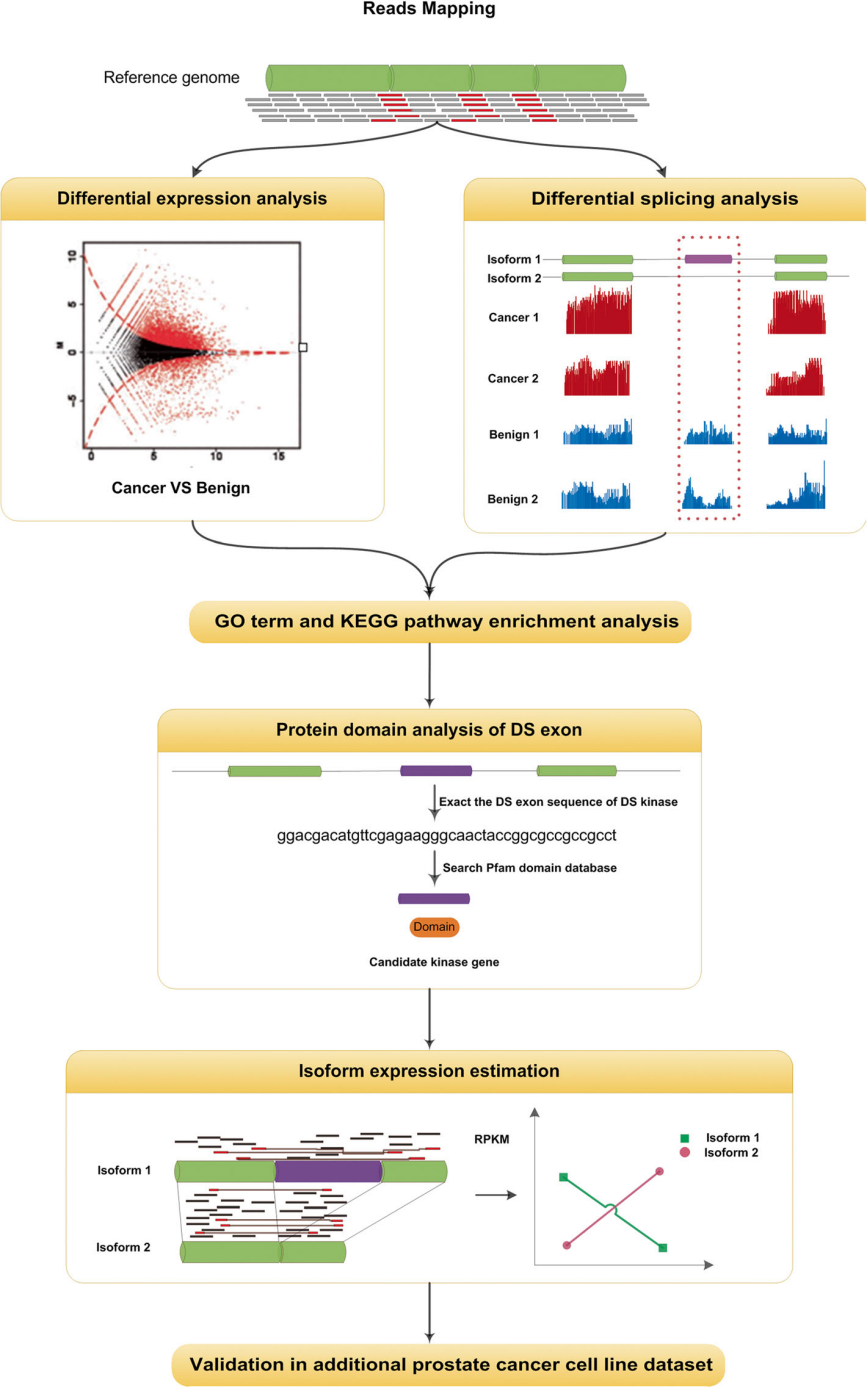
Overview of data analysis pipeline. After mapping the RNA-seq reads to the reference genome, differential expression (DE) analysis and differential splicing (DS) analysis were applied on the estimated gene and exon expression values, using DEGseq [15] and DSGseq [16] plus DEXseq [17], respectively. Enrichment analysis on GO terms and KEGG pathways were applied on the obtained lists of DE genes and DS genes to get the overall picture of functions of the DE and DS genes. Protein domain analysis was applied on the DS exons of the kinase genes. Isoform expressions were estimated using NURD [20] to quantitatively study the isoform proportions of candidate genes. An additional dataset was used to validate the observations

To characterize the transcriptome profile in prostate cancer, we analyzed an RNA-Seq dataset by Kannan et al. [12] using the strategy we discussed in [4]. The pipeline of the analysis is shown in Fig. 1. The RNA-Seq data of 20 prostate cancer and ten matched benign tissue samples were downloaded from NCBI SRA database (accession number SRP002628). Another prostate cancer cell line dataset was obtained for validation from SRA (accession number SRP004637), which contain 58 RNA-Seq samples from 21 prostate cancer cell lines [13]. The short sequencing reads were first remapped to human reference genome using TopHat (version 1.4.1) [14]. Uniquely mapped reads with no more than two mismatches were kept for downstream analyses.

Identification of DE genes was conducted using DEGseq (version 1.2.2) [15]. Genes were reported as differentially expressed for downstream analysis at two significance cut-offs: *p*-value < 0.05 and FDR < 1%.

We used DSGseq [16] and DEXSeq (version 1.6.0) [17] to detect DS genes between the two groups. DSGseq uses negative-binomial distribution to model read counts on exons and defines a NB-statistic to detect differences in the usage of exons between two groups. The cut-off of NB_stat = 2 was used, corresponding to a moderate stringency [16]. DEXSeq also detects differential splicing in an exon-centric manner with *p*-values reported for differential usage of each exon. We chose adjusted *p*-value < 0.05 as cut-off for DS exons. The consensus results of the two methods were reported as DS exons and DS genes.

We used GOseq (version 1.10.0) [18] to identify Gene Ontology (GO) terms which are over-represented in the detected genes. GOseq has special designs in its statistical test for avoiding possible selection bias toward long and highly expressed genes. GO terms were considered as statistically significant with false discovery rate (FDR) < 0.05. We mapped the DE and DS kinase genes to KEGG pathways and used the DAVID tool [19] to study pathways they are enriched in. Pathways with *p*-value < 0.05 were declared significant.

Pfam domain search was carried out to analyze the protein kinase activity of the alternative splicing exons in the DS kinase genes. We built a background set by extracting all alternative exons of 518 kinase genes according to the annotation of hg18 downloaded from UCSC Genome Browser. DNA regions of both the DS exons and the background set were translated into peptides in six-frame manner using sequence translation tool “EMBOSS Transeq”. The peptides sequences were searched against Pfam database for matching domains with the default cut-off value of E-value = 1.0. Fisher’s Exact Test was performed to compare the kinase domain hits of DS kinase exons and background exons (All the customized scripts are available upon request).

The software NURD (version 1.1.0) [20] was used to study the isoform expression levels of DS genes. It uses nonparametric models to deal with possible position-related biases in RNA-Seq data and estimates the isoform expression by maximizing a likelihood function.

## Results

### Differential expression analysis reveals gene signatures of prostate cancer

Comparing prostate cancer and benign tissue samples with DEGseq, we identified 3093 genes as differentially expressed with *p*-value < 0.05. Among them, 528 genes are differentially expressed with FDR < 1% (Additional file 1). Focusing on kinase genes, we found 62 DE kinase genes at *p*-value < 0.05 and 16 DE kinase genes at FDR < 1% (Additional file 2).

We listed the top 10 up-regulated genes and top 10 down-regulated genes and their function annotations in Table 1. Most of the top genes have been reported to be related with prostate cancer. The top up-regulated gene in prostate cancer is phospholipase A2 group (PLA2G2A), an important enzyme in inflammatory processes. The expression of PLA2G2A has been reported significantly increased in prostate cancer comparing to benign tissue, and it might serve as a prognostic maker for prostate cancer [21]. The second up-regulated gene orosomucoid 1 (ORM1) is an AR-activated gene and involved in AR pathway [22]. Ayla et al. found that ORM1 was differentially expressed between non-recurrent primary and metastatic prostate cancer and was involved in cancer metabolism and immune response pathways [23]. Notably, the fifth up-regulated gene antigen 3 (PCA3) is a non-protein coding gene. PCA3 is prostate-specific and has been shown highly expressed in prostate cancer, and has been identified as a genetic marker for prostate cancer diagnosis [24].

**Table 1:** Top 10 up-regulated and down-regulated genes in prostate cancer samples

Gene Symbol	log2(Fold-change)	*p*-value	Role in prostate cancer based on literature and database search
**Top 10 up-regulated genes**			
PLA2G2A	2.3884419	0	An enzyme in inflammatory process, might serve as a prognostic marker
ORM1	3.9940791	0	AR-activated gene and involved in AR pathway
TARP	2.0779529	3.13E-196	Prostate-specific protein and leads to increased growth rate of prostate cancer cells
ORM2	3.0434879	1.52E-157	
PCA3	4.0035279	1.26E-132	Highly expressed in prostate cancer tissue, a genetic marker for prostate cancer diagnosis
SPON2	2.0706815	9.75E-121	Over expressed in prostate cancer tissues, could be used as a biomarker
HPN	2.4321643	5.79E-49	Involved in maintenance of cell morphology and growth of various cancer types, correlates with progression of prostate cancer and promotes cancer progression and metastasis
STEAP4	1.9395011	4.48E-44	Module metastasis and invasion of cancer cells through regulating focal adhesion kinase activation
MCCC2	2.0394019	4.89E-41	AR-regulated gene and strongly up-regulated in primary prostate cancer
PDLIM5	1.8980125	2.39E-40	
**Top 10 down-regulated genes**			
SERPINA3	−1.496602	0	Associated with increased risk of prostate cancer
LTF	−1.576865	0	Plays roles in several important biological processes such as cellular growth, inflammatory response, cancer development and metastasis
SAA1	−4.021895	0	
LCN2	−2.156207	0	
SOD2	−0.886676	1.82E-268	A tumor suppressor gene, the suppression of prostate cancer cell growth by mediating the senescence-associated tumor suppression
CFB	−1.251122	5.91E-225	
MT2A	−0.516269	2.57E-188	Interact with kinase domain of protein kinase Cmu(PKCmu) in prostate cancer
OLFM4	−1.481793	6.63E-188	Inhibit prostate cancer growth and metastasis with nteraction of cathepsin D and SDF-1
PSCA	−0.367134	1.70E-186	
S100A9	−2.169907	8.63E-143	

The most down-regulated gene in prostate cancer is serpin peptidase inhibitor, clade A (alpha-1 antiproteinase, antitrypsin) member 3(SERPINA3), whose protein product Alpha 1 antichymotrypsin (ACT) is associated with increased risk of prostate cancer [25]. The second down-regulated gene lactotransferrin (LTF) is a member of transferrin family and its protein product is a major iron-binding protein. One study identified LTF as the most significantly down-regulated gene in prostate cancer cells and proved that LTF protein can inhibit the growth of prostate cancer cells [26]. Its down regulation might be involved in prostate cancer progression [26]. Another gene that deserves highlight is superoxide dismutase 2(SOD2). It encodes a mitochondrial enzyme that can protect cell from oxidative damage, and has been known as a tumor suppressor gene in human prostate cancer [27]. Increased expression of SOD2 can result in the suppression of prostate cancer cell growth by mediating the senescence-associated tumor suppression with insulin-like growth factor binding protein related protein-1 (IGFBP-rP1) [27]. The down regulation of SOD2 may help promote the cancer progression.

### Most differentially spliced genes in prostate cancer do not show differential total expression

Besides differential expression analysis at gene level, we studied possible splicing variations between cancer and benign tissues. Using DSGseq and DEXseq, we identified 2651 genes that are differentially spliced between prostate cancer and benign prostate tissues (Additional file 3). This includes 55 DS kinase genes, each of which has one differentially spliced exon (Additional file 4).

**Fig. 2:**
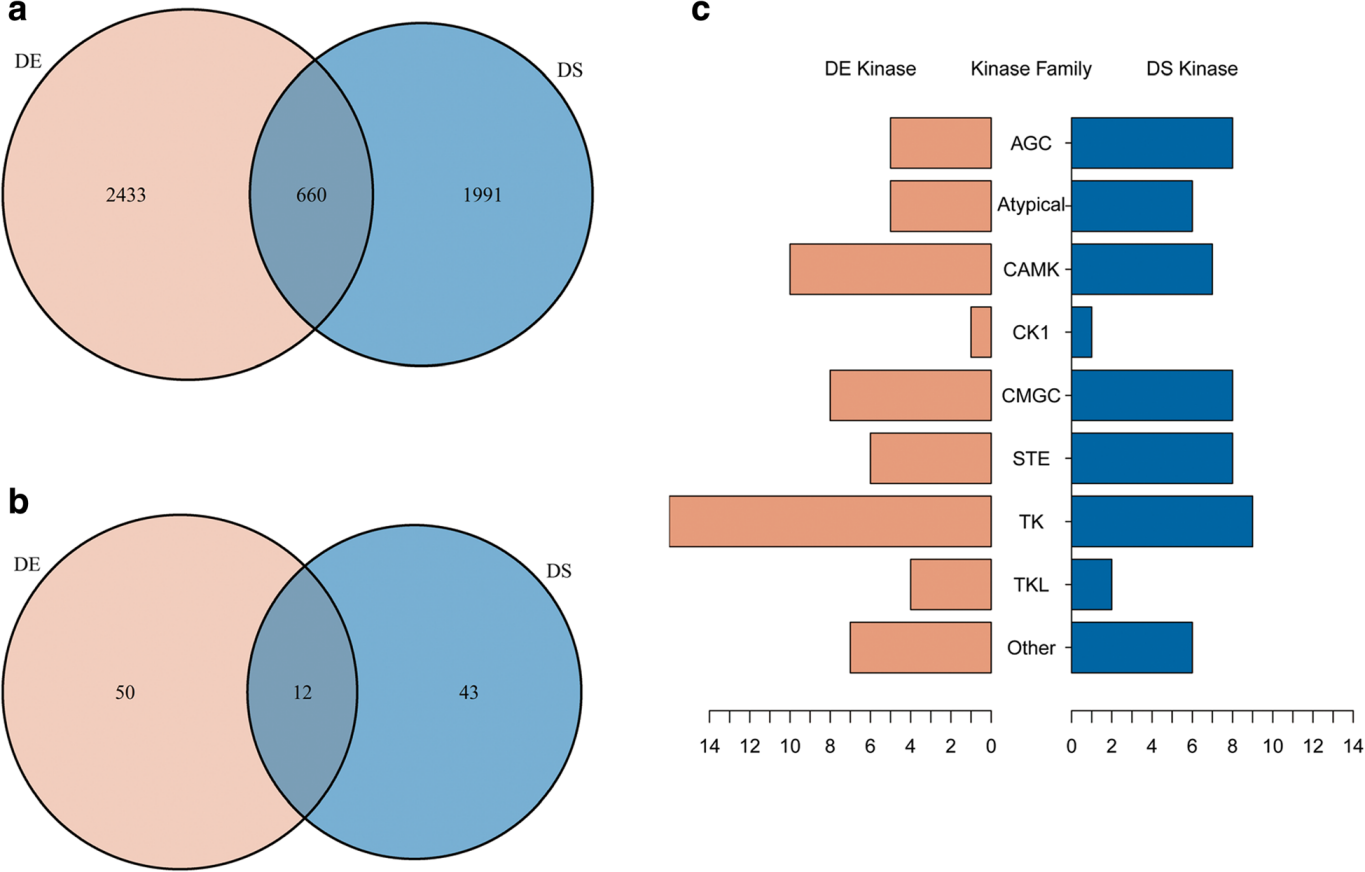
Profiles of DE and DS genes between prostate cancer and benign tissues. **a** Overlap of differential expression (DE) and differential splicing (DS) genes. **b** Overlap of DE and DS kinase genes. **c** Counts of DE and DS kinase genes in protein kinase families

We compared the profiles of DS and DE genes. The overlap of two profiles has 660 genes (Fig. 2a), composing less than 25% of the DE genes. Among the 55 DS kinase genes, only 12 are differentially expressed between cancer and benign samples at gene level (Fig. 2b). Most DS kinases tend to have stable overall expression level but have different splicing isoforms and different exon usages. The small overlap between differential expression and differential splicing indicates that gene expression regulation can be modulated at both gene-level and isoform-level. Different isoform ratios and different major isoforms can have significant functional impact. The detected DE or DS kinase genes belong to nine eukaryotic kinase families. The tyrosine kinase (TK) family has the largest number of members (Fig. 2c). Tyrosine kinase can phosphorylate tyrosine amino acid on substrates specifically. TK members function in a wide variety of processes and pathways, from transmembrane signaling, signal transduction to the nucleus, to cell-cycle control and transcription factor activation [28]. TK has also been found associated with several cancers and have implications for cancer treatment [29].

### GO and KEGG enrichment analysis show distinct functions of DE and DS genes in prostate cancer

We performed GO and KEGG pathway enrichment analysis on the detected DE and DS genes to investigate their biological relevance to prostate cancer. GO terms related with cancer and prostate cancer like cell proliferation, cell adhesion, and prostate gland development are found to be enriched by DE genes (Additional file 5: Figure S1 and Additional file 6). Enriched GO terms and KEGG pathways of DE genes include ribosome, metabolic pathways, p53 signaling pathway, MAPK signaling pathway and other disease-related pathways of Parkinson’s disease, Alzheimer’s disease, prostate cancer and bladder cancer, etc. (Additional file 5: Figure S2 and Additional file 6). Three genes closely related to AR functions in prostate cancer, AR, KLK3 and MAPK3, are among the detected DE genes. In comparison, GO enrichment analysis of DS genes shows a wide range of GO terms related to mRNA metabolism, regulation of catalytic activity, intracellular transport, and protein complex subunit organization etc. (Additional file 5: Figure S3). Pathways including proteasome, ribosome, and spliceosome are found to be enriched by DS genes (Additional file 6).

**Fig. 3:**
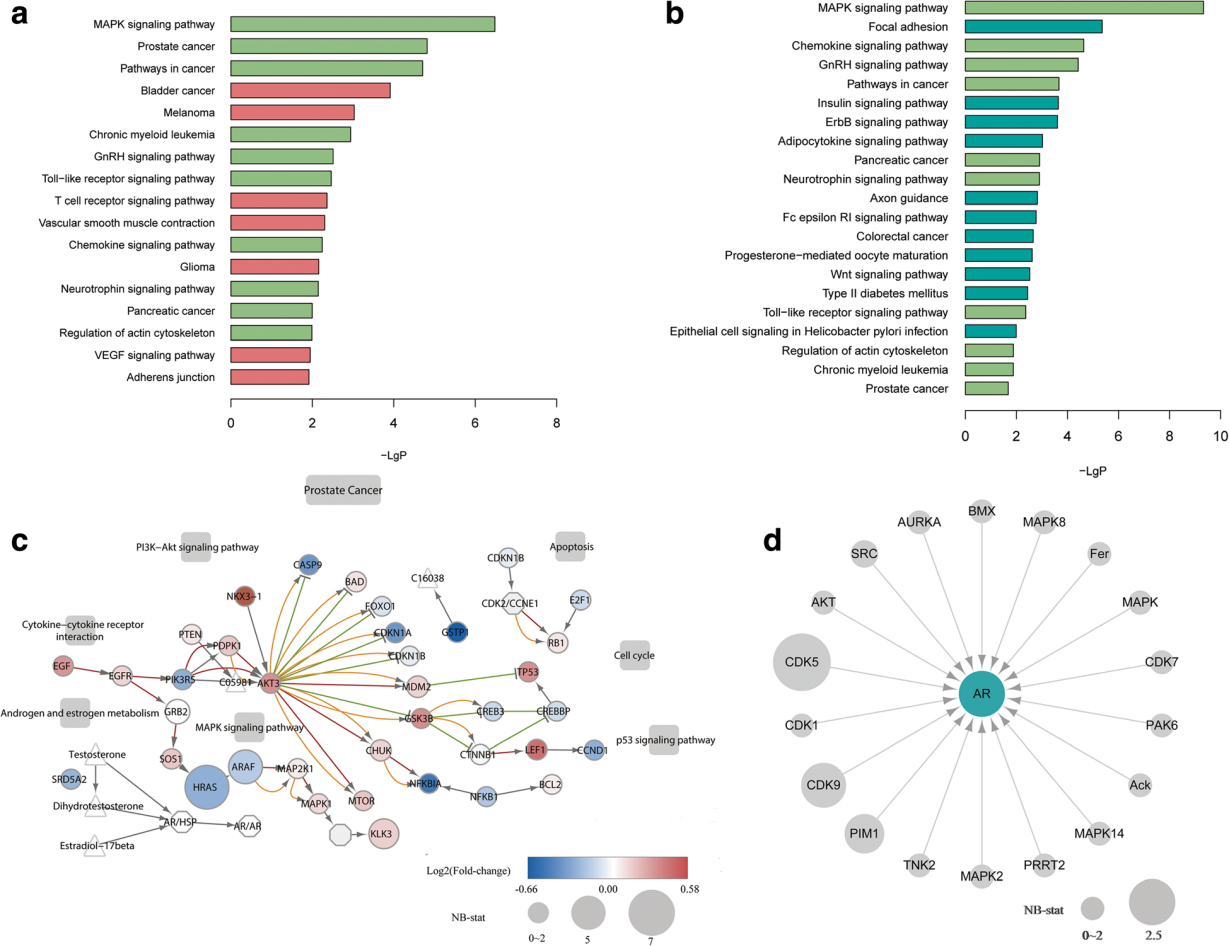
Functional enrichment analysis of DE and DS kinase genes. **a** Enriched KEGG pathways of DE kinase genes. **b** Enriched KEGG pathways of DS kinase genes. Pathways enriched in both DE and DS kinase genes are shown in light green in both plots. Those only enriched in DE kinase genes are shown in purple in (**a**), and those only enriched in DS kinase genes are shown in dark green in (**b**). **c** The “Prostate Cancer” pathway in KEGG, with detected DE and DS genes highlighted in colors. Circles represent genes, with their colors showing their fold-change between the cancer and normal groups. Circle size indicates the NB-stat calculated by DSGseq, which represents the degree of differential splicing of the gene between the two groups. Small molecules (triangles), cellular process (rectangles) and gene complexes (hexagon) are also shown. Colored edges indicate activation (red), inhibition (green), phosphorylation (orange). **d** Expression and splicing changes of kinases that phosphorylate AR. None of the kinase genes is differentially expressed, but CDK5, CDK9, PIM1 and SRC are differentially spliced, with node sizes representing the NB-stat in DSGseq

We also did functional enrichment analysis of the DE and DS kinases comparing to the background of all kinases. Interestingly, enriched GO terms of DE kinases can be summarized as negative regulation of cellular metabolism, enriched GO terms of DS kinases can be summarized as positive regulation of biological process and protein metabolic process, and both DE and DS kinases are found enriched in GO terms related to protein phosphorylation (Additional file 5: Figure S4 and Additional file 7). In the KEGG pathway analysis, detected DE kinases show significant enrichment in cancer-related pathways (Fig. 3a, Additional file 7), with the MAPK signaling pathway and prostate cancer at the top of the list. The MAPK pathway also comes at the top of the list enriched by DS kinase genes, followed by focal adhesion etc. (Fig. 3b).

To visualize the DE and DS gene profiles in a context of pathways, we compiled the KEGG pathway map “Prostate Cancer” by considering the expression and splicing difference between cancer and benign (Fig. 3c). The gene KLK3 encodes the glyco-protein enzyme known as prostate-specific antigen (PSA), an important tumor marker used for diagnosis of prostate cancer in clinical practices [24]. KLK3 is found to be both differentially expressed and spliced in the pathway. The gene HRAS was also found both differentially expressed and spliced. HRAS belongs to the Ras oncogene family, which encodes proteins functioning in signal transduction pathways. HRAS gene can produce two protein isoforms with complementary function related to cell proliferation [30]. The differential splicing of gene HRAS may suggest the roles of expression change at isoform level in prostate cancer. We noted that none of the kinases that can phosphorylate AR were differentially expressed, but four kinases, CDK5, CDK9, PIM1 and SRC were differentially spliced (Fig. 3d). Searching in the literature, we found that these four DS kinases had been reported to be involved in prostate cancer in multiple functions [31–34].

### Differentially spliced kinase exons are significantly enriched in protein kinase domain

Most of protein kinases are involved in critical cellular activities. Splicing variability of functional region such as ATP binding domain and kinase domain may directly initiate or contribute to cancer progression. Alternative splicing may regulate oncogenic kinase activity by generating isoforms that skip kinase domain or truncate kinase domain [9]. We investigated that whether the 55 differentially spliced exons of DS kinases have functional impact on kinase activity by searching domains against Pfam database. We found 16 of the DS exons have domain hits in the Pfam domain database. Among them, 9 DS exons include the protein kinase domain (Pkinase). To verify if the functional relevance of DS exons of the 55 DS exons to the kinase domain was more significant than random chance of all alternative splicing kinase exons, we collected all known alternative splicing exons of 518 kinases from UCSC Genome Browser as background and searched them in the Pfam database. In total, 81 significant Pkinase domains were detected in 8671 peptide sequences translated from 1936 alternative splicing exons of the 518 kinases. Comparing to this background, we found that the 55 DS exons we identified are significantly more related to protein kinase domains (*p*-value < 0.0007 by Fisher’s Exact Test). Besides, we investigated that whether the kinase domain predicted by Pfam search really existed in the annotation database GeneCards [35]. We found that either truncated kinase domain or total kinase domain exist in the DS exons of the nine kinases.

These results indicated that DS exons of the DS kinases are significantly enriched in kinase domains. Differential splicing of these kinases may modify kinase activity and have functional impact on phosphorylation of their substrates in prostate cancer progression by differential expression of isoforms including or excluding those DS exons.

### Isoform switching of CDK5 in prostate cancer and its potential functions on AR phosphorylation

**Fig. 4:**
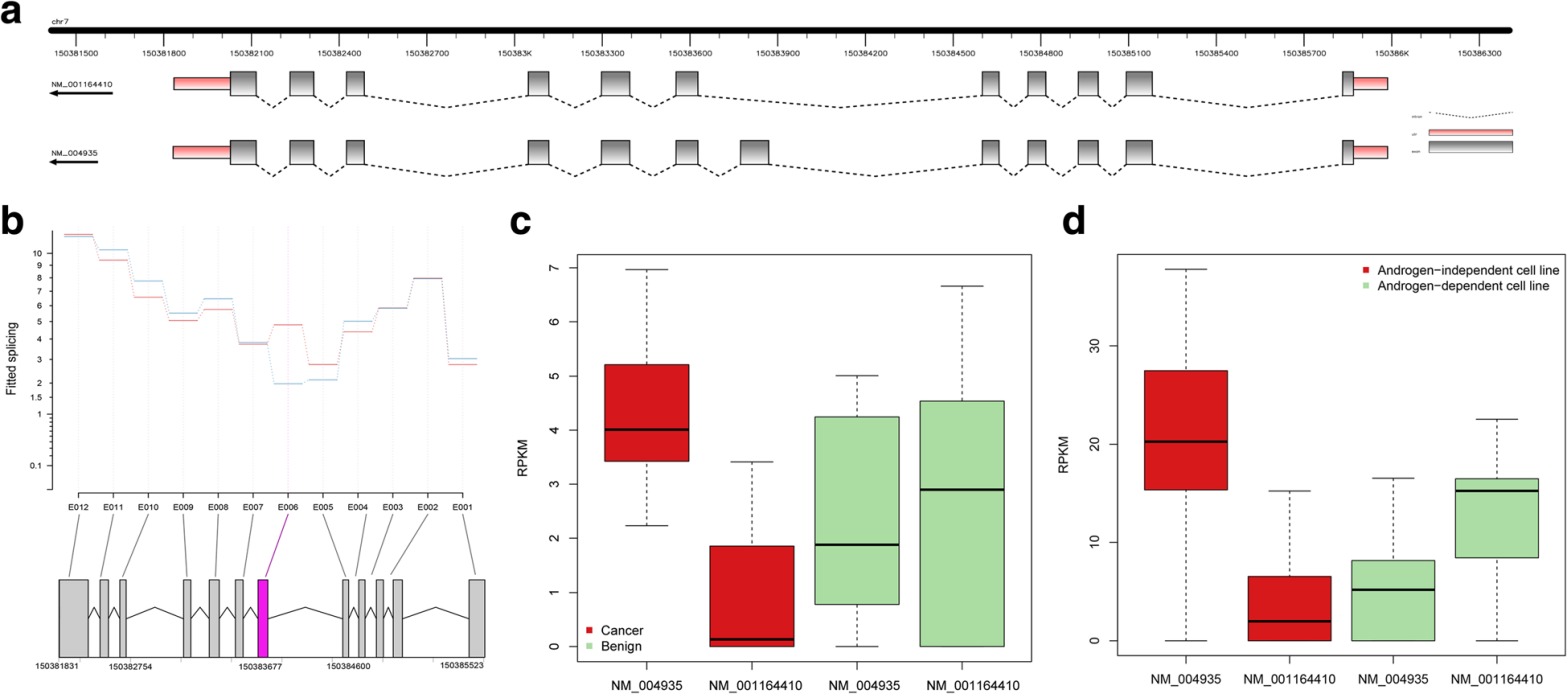
Differential splicing and isoform switching of kinase gene CDK5 in prostate cancer. **a** The transcript structure of CDK5. CDK5 is located on Chromosome 7 and has two annotated isoforms: NM_004935 (Isoform1) and NM_001164410 (Isoform2). Isoform1 has 12 exons, and its 6th exon is skipped in Isoform2. **b** Differential splicing of CDK5 illustrated by DEXSeq [17]. The relative exon usage, as measured by “fitted splicing”, was plotted for each exon in two groups. Blue lines are for prostate benign samples and red lines are for prostate cancer samples. The panel at the bottom shows the location of the exons, with the alternative exon highlighted in red. **c** Expression boxplots of the two isoforms in prostate cancer and benign tissue samples. **d** Expression boxplots of the two isoforms in the androgen-independent and androgen-dependent prostate cancer cell lines

Among the 9 DS exons on kinase domain, we found that the DS exon of CDK5 is included in one isoform and excluded in the other. We took CDK5 as an example to conduct further isoform-level analysis. The DS kinase gene CDK5 has two isoforms (Fig. 4a). We found that the two isoforms are differentially used between prostate cancer and benign tissues (Fig. 4b). The inclusion and exclusion of the 32 amino acid DS exon (exon 6) that defines the two isoforms, and this exon is differentially used between the cancer and normal samples. We refer the longer isoform with exon 6 to as Isoform1 (NM_00495 in REFSEQ), the shorter isoform as Isoform2 (NM_001164410).

Database searching revealed that the serine/threonine protein kinase active site lies in this alternative exon of gene CDK5 (Additional file 5: Figure S5). Isoform1 has full length of 293 amino acids and encodes protein CDK5 while Isoform2 lacks exon 6 and encodes a 260 amino acids protein. CDK5 is one of the cyclin-dependent kinase (CDK) family members and plays important roles in various cellular activities like cell differentiation and apoptosis [36]. The function of protein encoded by Isoform2 has not been well studied in the literature. Kim et al. reported different subcellular localizations of two isoforms of CDK5 which might indicate different functions [37]. CDK5 knock-down by siRNA resulted in changes of the microtubule cytoskeleton, loss of cellular polarity and motility in human prostate cancer DU 145 cell line [34]. It has been reported that protein CDK5 can activate and stabilize AR in the nucleus through Ser-81 phosphorylation in prostate cancer cells [38]. These studies all suggested that CDK5 may be a potential regulator in prostate cancer progression through AR phosphorylation.

Our study showed that CDK5 is differentially spliced between prostate cancer and benign tissues at exon 6 (Fig. 4b). We found that although CDK5 was not detected as a DE gene at gene expression level, the relative isoform abundances in prostate cancer and benign tissues are very different. Isoform1 makes up more than 90% of the total expression of CDK5 in prostate cancer but less than 40% in benign tissues (Fig. 4c, Additional file 8). AR shows higher expression in the prostate cancer group, with log2 fold change of ~ 1.43 comparing to the benign group. It has been reported that protein CDK5 encoded by Isoform1 can phosphorylate AR at its Ser-81 site and therefore activate and stabilize AR to promote prostate cancer cell growth in prostate cancer cell line [38]. Since the kinase domain is missed in Isoform2, this function would be lost or weakened when Isoform2 is dominate in normal prostate tissues. We inferred that the higher relative expression of CDK5 Isoform1 in cancer cells can plays an important role in AR-involved prostate cancer progression.

We validated the CDK5 isoform expression pattern in another RNA-Seq dataset of 21 prostate cancer and benign cell lines [13]. This dataset contains only five benign cell lines and the prostate cancer cell lines are of different subtypes. We observed that the benign cell lines still have lower Isoform1 expression (Additional file 8), although the isoform-switching between the prostate cancer and benign cell lines is statistically not significant. We found that isoform1 tends to be not expressed or lowly expressed in some prostate cancer cell lines like VCaP and LNCaP, which are androgen-dependent prostate cancer cell lines. We applied clustering analysis on the cell lines by their gene expression profiles, and clustered them into two groups: androgen-dependent cell lines and androgen-independent cell lines. We observed that CDK5 has different isoform usage between androgen-dependent and androgen-independent prostate cancer cell lines. Isoform1 takes ~ 81% of total expression in the androgen-independent group and ~ 28% in the androgen-dependent group, and the proportions of Isoform2 are ~ 19% and ~ 72%, respectively (Fig. 4d, Additional file 8). These different isoform preferences of CDK5 between androgen-independent or dependent prostate cancer cell lines implies that CDK5 can modulate AR transcriptional activity through differential splicing in accordance with the present or absent of androgen.

## Discussion

In this study, we characterized the gene expression profile of prostate cancer by a systematic comparison of RNA-Seq data of prostate cancer and benign tissues. Quantitative analysis was applied on both the gene expression levels and their alternative isoforms, with a special emphasis on kinase genes. Results showed that alternative splicing adds another layer of regulation to gene expression by the different usage of splicing isoforms or their different relative expressions in cancer. Functional enrichment analysis also showed that the differentially expressed and spliced genes are enriched in GO terms and KEGG pathways that are closely related to prostate cancer. Some kinase genes that can phosphorylate AR are differentially spliced but none of them are differentially expressed. Differentially spliced exons of detected kinase genes are more enriched in the protein kinase domain than other alternative splicing exons. This indicates that the differentially spliced kinases have the potential to alter protein phosphorylation activity by changing abundance of isoforms with kinase domain.

**Fig. 5:**
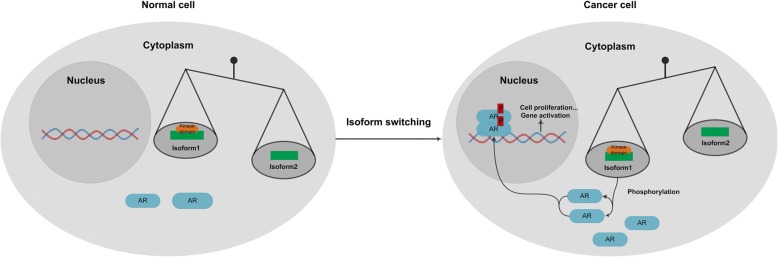
A hypothetic model of the function of CDK5 isoform switching in prostate cancer. The dominance of Isoform1 over Isoform2 in cancer increases the phosphorylation of AR, which increases its protein stabilization and activity in nucleus, and thus promote cancer-related cellular process like cell proliferation

This conclusion was well illustrated by the observation that the AR phosphorylation kinase gene CDK5 undergoes isoform switching between prostate cancer and benign tissues. This result was confirmed on another RNA-Seq dataset of prostate cancer cell lines, on which isoform ratio differences were observed between androgen-dependent and androgen-independent prostate cancer cell lines. We speculate that the isoform usage preference of CDK5 plays an important role in cancer cell growth by modulating AR transcriptional activity though AR phosphorylation and AR protein stabilization, as illustrated in the hypothetic model of Fig. 5. The isoform ratio change of CDK5 can act as a fine tuner of AR activity and contribute to prostate cancer progression.

## Conclusions

In summary, our study provided a transcriptome profile of prostate cancer using RNA-Seq data with an emphasis on differentially spliced kinase genes. It indicated alternative splicing has critical impact on kinase activity in cancers. Especially, isoform switching of kinase gene CDK5 was found in prostate cancer and benign tissues, which suggests its regulatory role in AR phosphorylation via alternative splicing. The work brings new understanding to the role of alternatively spliced kinases in prostate cancer and provides an example on the systematic analysis of RNA-Seq data in cancer.

## Additional files

Additional file 1: **Table S1.** DE genes with *p*-value < 0.05 and FDR < 0.01. (XLSX 448 kb)

Additional file 2: **Table S2.** DE kinase genes with *p*-value < 0.05 and FDR < 0.01. (XLSX 20 kb)

Additional file 3: **Table S3.** DS genes detected by both DSGseq and DEXseq. (XLSX 374 kb)

Additional file 4: **Table S4.** DS kinase genes detected by both DSGseq and DEXseq. (XLSX 18 kb)

Additional file 5: **Figure S1.** DE gene counts in prostate cancer related GO terms. **Figure S2.** Enriched KEGG pathways by DE genes. **Figure S3.** REVIGO treemap for enriched GO terms by DS genes. **Figure S4.** REVIGO treemap for enriched GO terms by DE and DS kinase genes. **Figure S5.** Kinase domain and phosphorylation site of CDK5 two protein isoforms. (PDF 836 kb)

Additional file 6: **Table S5.** GO and KEGG enrichment analysis results for DE and DS genes. (XLSX 220 kb)

Additional file 7: **Table S6.** GO and KEGG enrichment analysis for DE and DS kinase genes. (XLSX 29 kb)

Additional file 8: **Table S7.** CDK5 isoform expression in two datasets. (XLSX 17 kb)

## References

[1] Wang ET, Sandberg R, Luo S, Khrebtukova I, Zhang L, Mayr C (2008). Alternative isoform regulation in human tissue transcriptomes. Nature.

[2] Chen M, Manley JL (2009). Mechanisms of alternative splicing regulation: insights from molecular and genomics approaches. Nat Rev Mol Cell Biol.

[3] Ghigna C, Valacca C, Biamonti G (2008). Alternative splicing and tumor progression. Curr Genomics.

[4] Feng H, Qin Z, Zhang X (2013). Opportunities and methods for studying alternative splicing in cancer with RNA-Seq. Cancer Lett.

[5] Shen MM, Abate-Shen C (2010). Molecular genetics of prostate cancer: new prospects for old challenges. Genes Dev.

[6] Manning G (2002). The protein kinase complement of the human genome. Science.

[7] Zhang J, Yang PL, Gray NS (2009). Targeting cancer with small molecule kinase inhibitors. Nat Rev Cancer.

[8] Merkin J, Russell C, Chen P, Burge CB (2012). Evolutionary dynamics of gene and Isoform regulation in mammalian tissues. Science.

[9] Druillennec S, Dorard C, Eychène A (2012). Alternative splicing in oncogenic kinases: from physiological functions to Cancer. J Nucleic Acids.

[10] Robinson D, He F, Pretlow T, Kung HJ (1996). A tyrosine kinase profile of prostate carcinoma. Proc Natl Acad Sci.

[11] Kniazev I, Cheburkin I, Spikermann K, Peter S, Jenster G, Bangma KH, Karelin MI (2003). Gene expression profiles of protein kinases and phosphatases obtained by hybridization with cDNA arrays: molecular portrait of human prostate carcinoma. Mol Biol (Mosk).

[12] Kannan K, Wang L, Wang J, Ittmann MM, Li W, Yen L (2011). Recurrent chimeric RNAs enriched in human prostate cancer identified by deep sequencing. Proc Natl Acad Sci.

[13] Prensner JR, Iyer MK, Balbin OA, Dhanasekaran SM, Cao Q, Brenner JC (2011). Transcriptome sequencing across a prostate cancer cohort identifies PCAT-1, an unannotated lincRNA implicated in disease progression. Nat Biotechnol.

[14] Trapnell C, Pachter L, Salzberg SL (2009). TopHat: discovering splice junctions with RNA-Seq. Bioinformatics.

[15] Wang L, Feng Z, Wang X, Wang X, Zhang X (2009). DEGseq: an R package for identifying differentially expressed genes from RNA-seq data. Bioinformatics.

[16] Wang W, Qin Z, Feng Z, Wang X, Zhang X (2013). Identifying differentially spliced genes from two groups of RNA-seq samples. Gene.

[17] Anders S, Reyes A, Huber W (2012). Detecting differential usage of exons from RNA-seq data. Genome Res.

[18] Young MD, Wakefield MJ, Smyth GK, Oshlack A. Gene ontology analysis for RNA-seq: accounting for selection bias. Genome Biol. 2010;11(2):R14. 10.1186/gb-2010-11-2-r14 PMC2872874 20132535

[19] Dennis G, Sherman BT, Hosack D, Yang J, Gao W, Lane HC, et al. DAVID: Database for Annotation, Visualization, and Integrated Discovery. Genome Biol. 2003;4(5):P3. PMC193660 12734009

[20] Ma X, Zhang X (2013). NURD: an implementation of a new method to estimate isoform expression from non-uniform RNA-seq data. BMC Bioinformatics.

[21] Mirtti T, Laine VJO, Hiekkanen H, Hurme S, Rowe O, Nevalainen TJ (2009). Group IIA phospholipase A2as a prognostic marker in prostate cancer: relevance to clinicopathological variables and disease-specific mortality. APMIS.

[22] Whitworth H, Bhadel S, Ivey M, Conaway M, Spencer A, Hernan R, et al. Identification of kinases regulating prostate Cancer cell growth using an RNAi phenotypic screen. PLoS One. 2012;7(6):e38950. 10.1371/journal.pone.0038950 PMC3384611 22761715

[23] Ergün A, Lawrence CA, Kohanski MA, Brennan TA, Collins JJ. A network biology approach to prostate cancer. Mol Syst Biol. 2007;3:82. 10.1038/msb4100125 PMC1828752 17299418

[24] Hessels D, Schalken JA (2009). The use of PCA3 in the diagnosis of prostate cancer. Nat Rev Urol.

[25] Christensson A, Björk T, Nilsson O, Dahlén U, Matikainen MT, Cockett AT (1993). Serum prostate specific antigen complexed to alpha 1-antichymotrypsin as an indicator of prostate cancer. J Urol.

[26] Shaheduzzaman S, Vishwanath A, Furusato B, Cullen J, Chen Y, Bañez L (2007). Silencing of lactotransferrin expression by methylation in prostate cancer progression. Cancer Biol Ther.

[27] Plymate SR, Haugk KH, Sprenger CC, Nelson PS, Tennant MK, Zhang Y (2003). Increased manganese superoxide dismutase (SOD-2) is part of the mechanism for prostate tumor suppression by Mac25/insulin-like growth factor binding-protein-related protein-1. Oncogene.

[28] Hubbard SR, Till JH (2000). Protein tyrosine kinase structure and function. Annu Rev Biochem.

[29] Paul MK, Mukhopadhyay AK (2004). Tyrosine kinase – role and significance in Cancer. Int J Med Sci.

[30] Guil S, De La Iglesia N, Fernández-Larrea J, Cifuentes D, Ferrer JC, Guinovart JJ (2003). Alternative splicing of the human proto-oncogene c-H-ras renders a new Ras family protein that trafficks to cytoplasm and nucleus. Cancer Res.

[31] Gordon V, Bhadel S, Wunderlich W, Zhang J, Ficarro SB, Mollah SA (2010). CDK9 regulates AR promoter selectivity and cell growth through serine 81 phosphorylation. Mol Endocrinol.

[32] Ha S, Iqbal NJ, Mita P, Ruoff R, Gerald WL, Lepor H (2012). Phosphorylation of the androgen receptor by PIM1 in hormone refractory prostate cancer. Oncogene.

[33] Saini S, Majid S, Shahryari V, Tabatabai ZL, Arora S, Yamamura S (2014). Regulation of SRC kinases by microRNA-3607 located in a frequently deleted locus in prostate Cancer. Mol Cancer Ther.

[34] Strock CJ (2006). Cyclin-dependent kinase 5 activity controls cell motility and metastatic potential of prostate Cancer cells. Cancer Res.

[35] Rebhan M, Chalifa-Caspi V, Prilusky J, Lancet D (1998). GeneCards: a novel functional genomics compendium with automated data mining and query reformulation support. Bioinformatics.

[36] Dhavan R, Tsai L-H (2001). A decade of CDK5. Nat Rev Mol Cell Biol.

[37] Kim T, Law V, Rosales JL, Lee K-Y (2010). Cdk5 variant 1 (cdk5-v1), but not full-length cdk5, is a centrosomal protein. Cell Cycle.

[38] Hsu F-N, Chen M-C, Chiang M-C, Lin E, Lee Y-T, Huang P-H (2011). Regulation of androgen receptor and prostate Cancer growth by cyclin-dependent kinase 5. J Biol Chem.

