# Supplementary material for: Characterization of kinase gene expression and splicing profile in prostate cancer with RNA-Seq data
Source: BMC Genomics. 2018 Aug 13;19(Suppl 6):564. doi: 10.1186/s12864-018-4925-1 (PMC6101066; doi:10.1186/s12864-018-4925-1)
Supplement: Supplementary file 5 — Figure S1. DE gene counts in prostate cancer related GO terms. Figure S2. Enriched KEGG pathways by DE genes. Figure S3. REVIGO treemap for enriched GO terms by DS genes. Figure S4. REVIGO treemap for enriched GO terms by DE and DS kinase genes. Figure S5. Kinase domain and phosphorylation site of CDK5 two protein isoforms. (PDF 836 kb) [file 12864_2018_4925_MOESM5_ESM.pdf]

Additional file 1

Figure S1

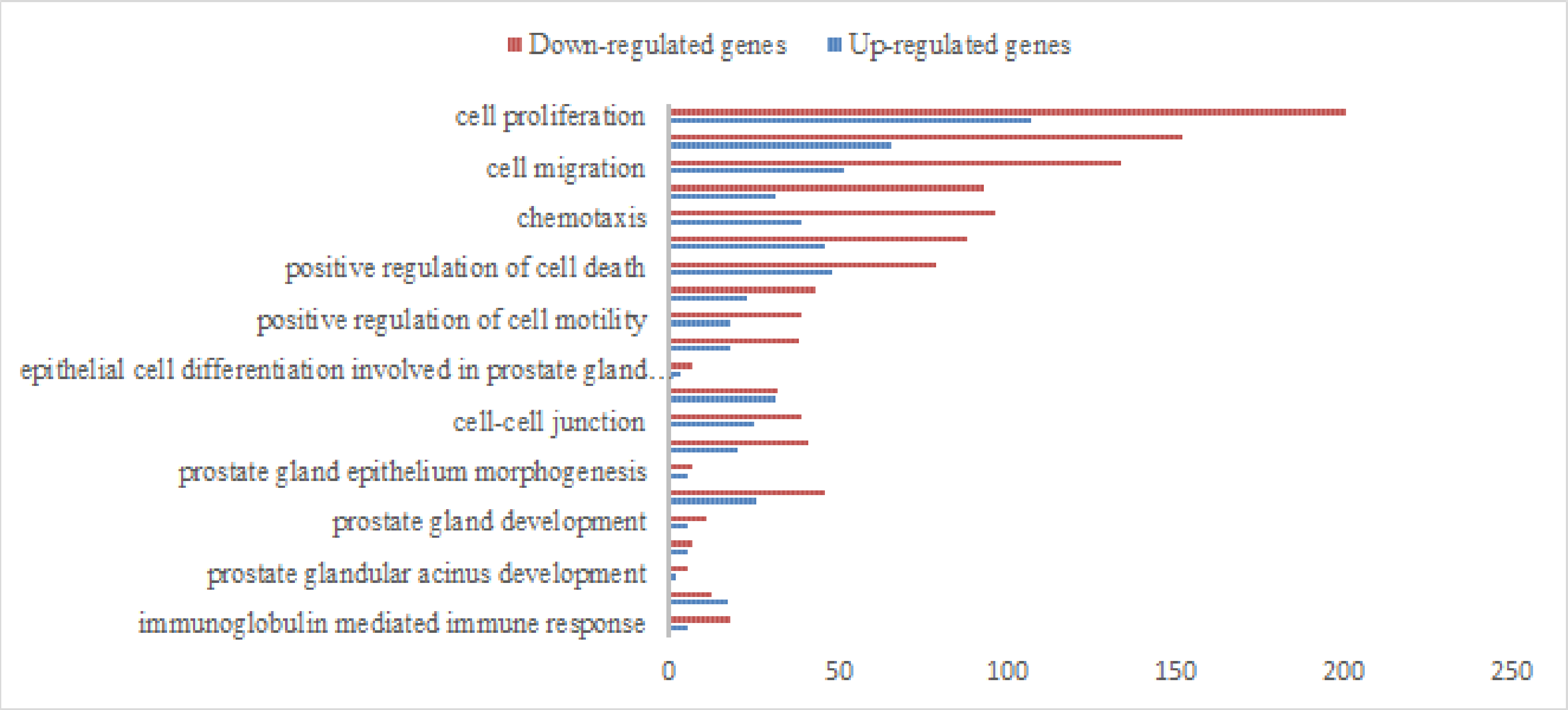

Figure S1: DE gene counts in prostate cancer related GO terms

Figure S2

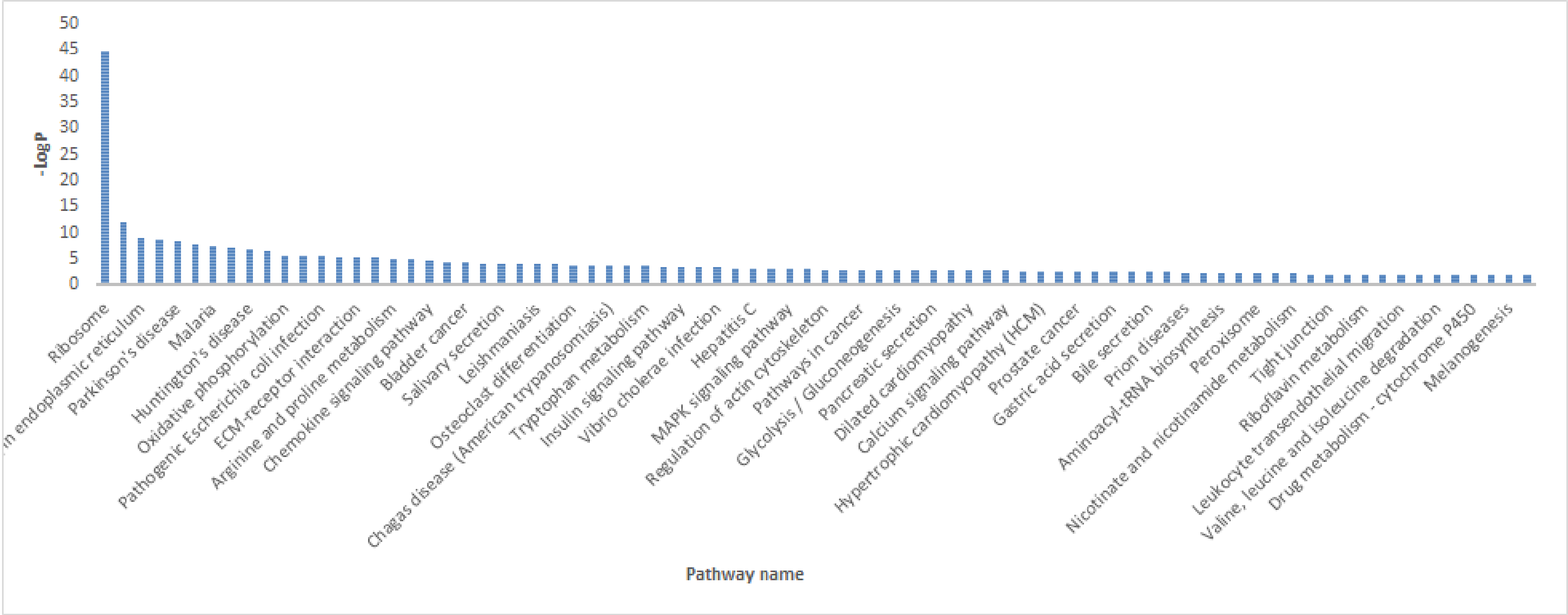

Figure S2: Enriched KEGG pathways by DE genes.

Figure S3

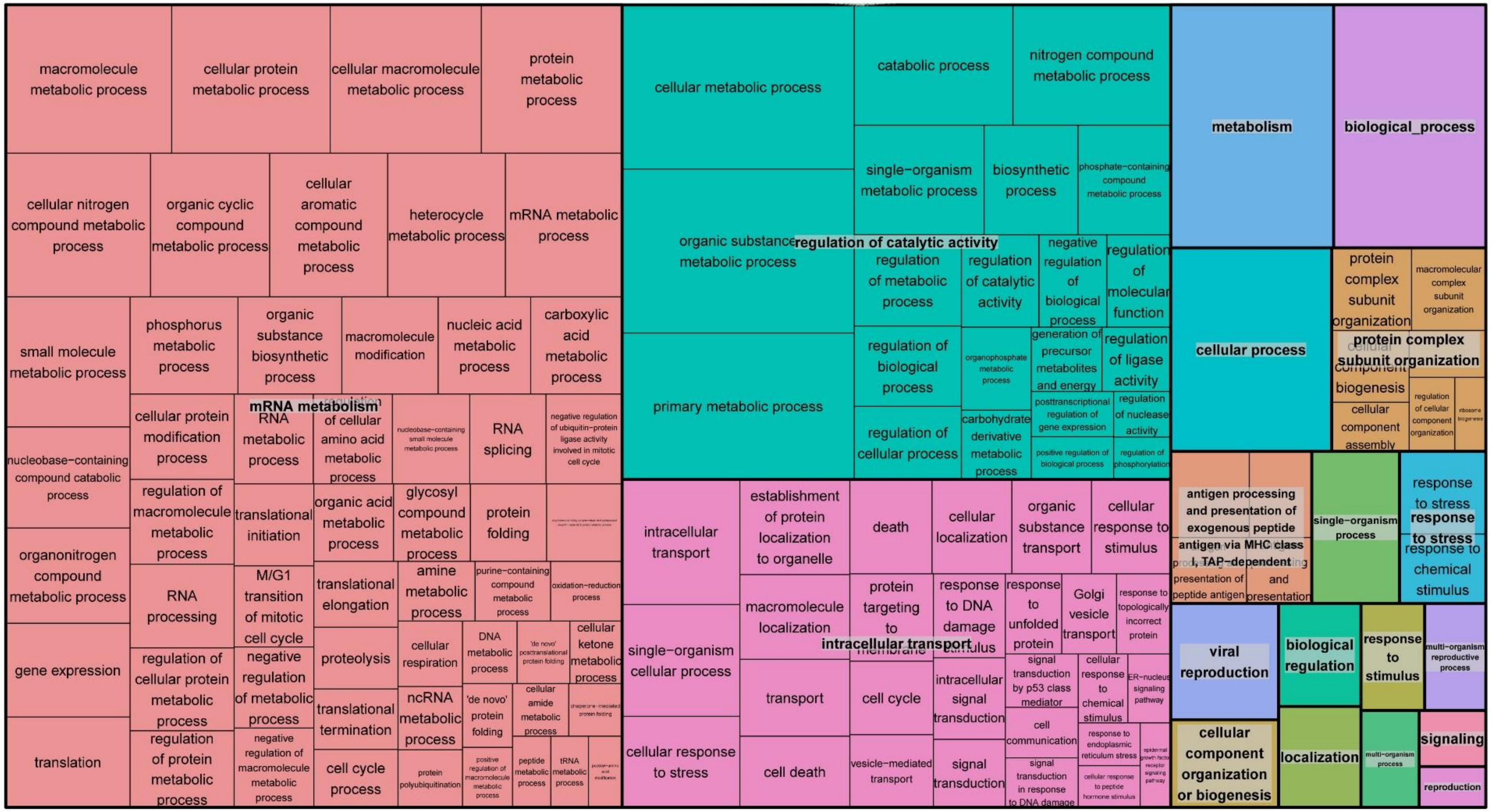

Figure S3: REVIGO treemap for enriched GO terms by DS genes. Here each rectangle is a cluster representative of loosely related GO terms.

Size of the clusters reflects the enriched p-value of GO terms. mRNA metabolism, regulation of catalytic activity, intracellular transport, protein complex subunit organization etc can be found enriched by DS genes.

Figure S4

A

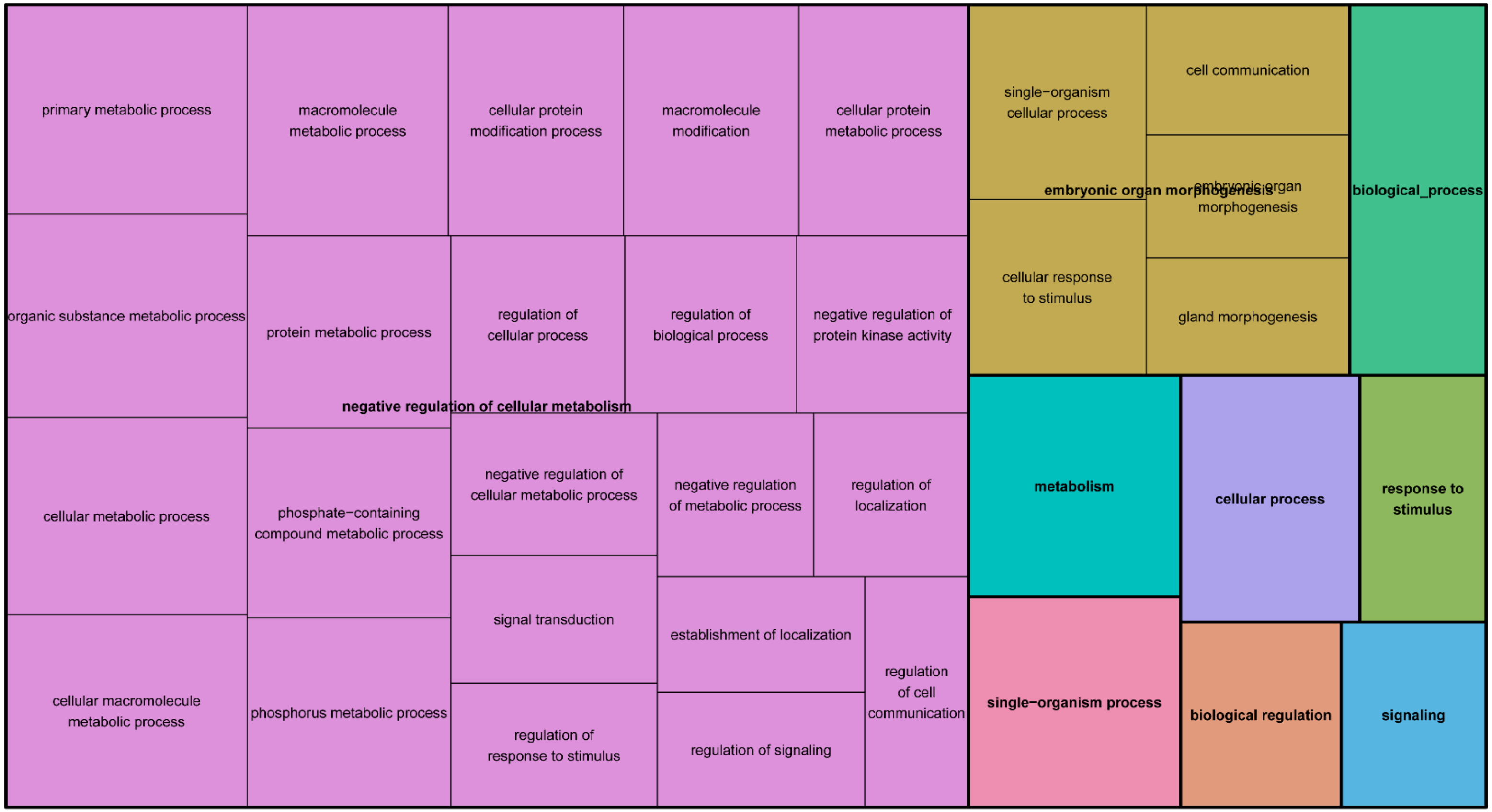
primary metabolic process
macromolecule metabolic process
cellular protein modification process
macromolecule modification
cellular protein metabolic process
single−organism cellular process
cell communication
embryonic organ morphogenesis
embryonic organ morphogenesis
biological_process
organic substance metabolic process
protein metabolic process
regulation of cellular process
regulation of biological process
negative regulation of protein kinase activity
cellular response to stimulus
gland morphogenesis
negative regulation of cellular metabolism
cellular metabolic process
phosphate−containing compound metabolic process
negative regulation of cellular metabolic process
negative regulation of metabolic process
regulation of localization
metabolism
cellular process
response to stimulus
signal transduction
establishment of localization
regulation of cell communication
cellular macromolecule metabolic process
phosphorus metabolic process
regulation of response to stimulus
regulation of signaling
single−organism process
biological regulation
signaling

B

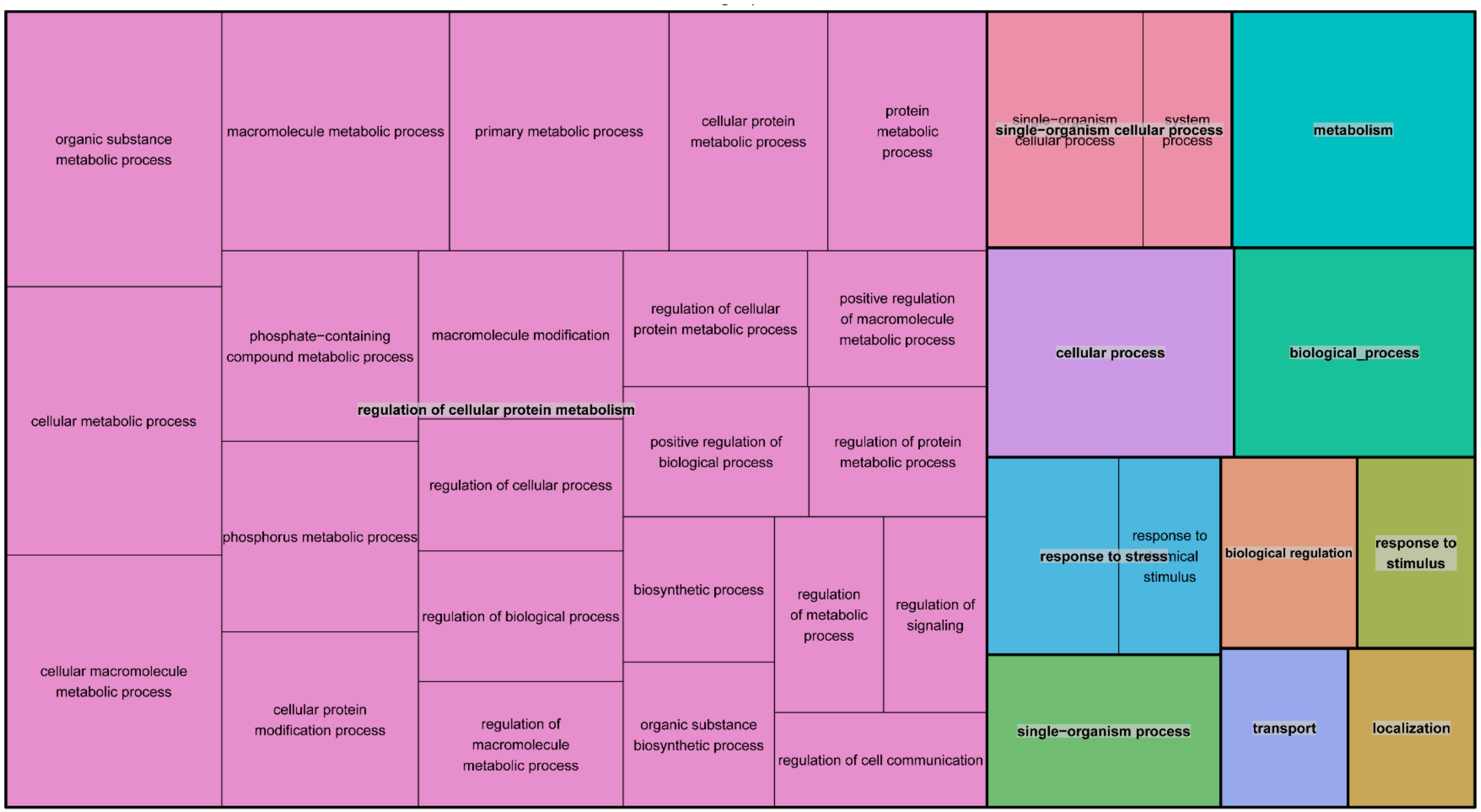

Figure S4: REVIGO treemap for enriched GO terms by DE (A) and DS (B) kinase genes. Here each rectangle is a cluster representative of loosely related GO terms. Size of the clusters reflects the enriched p-value of GO terms. Enriched clusters of DE kinases and DS kinases can be found to be opposite to each other. Enriched GO terms of DE kinases can be summarized into negative regulation of cellular metabolism including negative regulation of cellular metabolic process and metabolic process, while DS kinases are enriched in GO terms summarized by regulation of cellular protein metabolism including positive regulation of biological process and protein metabolic process.

Figure S5

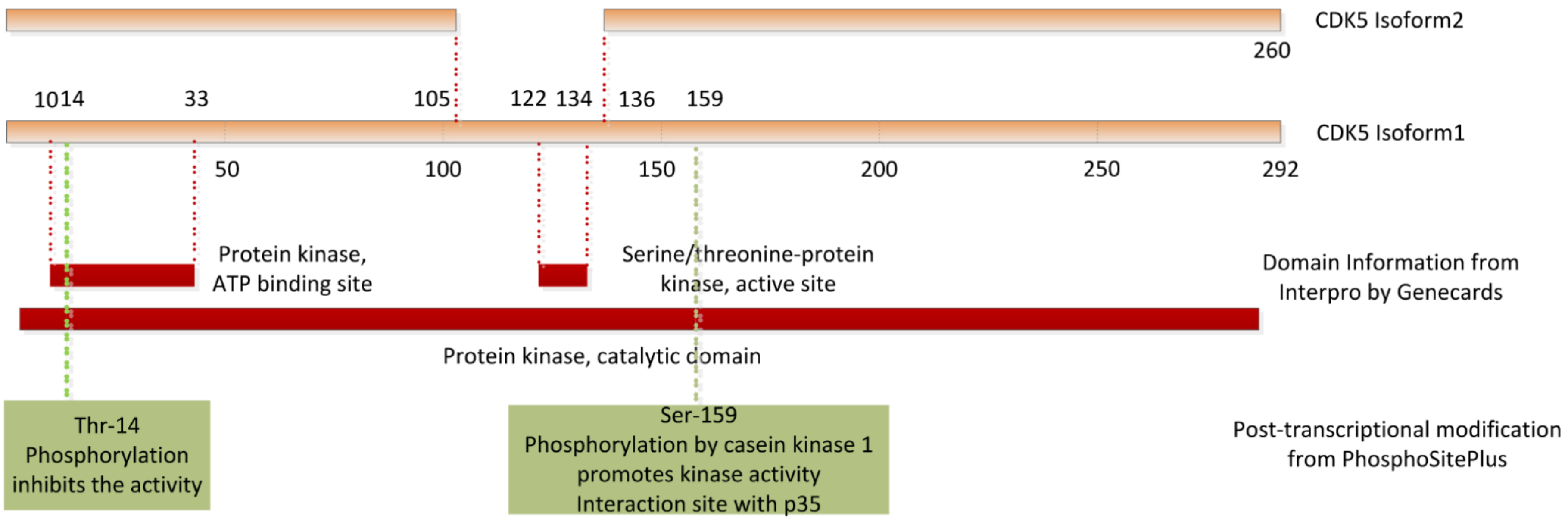

Figure S5: Kinase domain and phosphorylation site of CDK5 two protein isoforms. Exon6 of gene CDK5 encodes 32 amino acids from 105 to 136. A serine/threonine protein kinases active sit lies in this region. Here orange represents the isoforms of CDK5. Red represents the domain information from database. Green represents the post-transcriptional modification sites.
